# Physical Activity Patterns and Cardiometabolic Risk in Children and Adolescents with Obesity: A Cross-Sectional Study

**DOI:** 10.3390/diagnostics16081162

**Published:** 2026-04-14

**Authors:** Lorena Mihaela Manole, Elena Țarcă, Elena-Lia Spoială, Laura Otilia Boca, Mădălina Andreea Donos, Gabriela Păduraru, Gabriela Ghiga, Viorel Țarcă, Alin Constantin Pînzariu, Laura Mihaela Trandafir

**Affiliations:** 1 Grigore T. Popa University of Medicine and Pharmacy, 700115 Iași, Romania; lorena.manole@umfiasi.ro (L.M.M.); elena-lia.spoiala@umfiasi.ro (E.-L.S.); laura.boca@umfiasi.ro (L.O.B.); madalina.donos@umfiasi.ro (M.A.D.); paduraru.gabriela@umfiasi.ro (G.P.); gabriela.ghiga@umfiasi.ro (G.G.); alin.pinzariu@umfiasi.ro (A.C.P.); laura.trandafir@umfiasi.ro (L.M.T.); 2 Sfânta Maria Emergency Children Hospital, 700309 Iași, Romania; 3 Faculty of Medicine, Apollonia University, 700511 Iași, Romania; viorel.tarca@univapollonia.ro

**Keywords:** child, adolescent, obesity, physical activity, cardiometabolic risk, wearable devices, sedentary behavior, digital health

## Abstract

**Introduction**: Childhood and adolescent obesity is a growing global health challenge associated with early metabolic and cardiovascular complications. This study aims to compare questionnaire-assessed physical activity patterns and lifestyle characteristics among children and adolescents with obesity and normal-weight peers and to explore their associations with clinical measurements and cardiometabolic risk. Assessing resting metabolic rate (RMR) by indirect calorimetry may provide additional insight into metabolic status beyond conventional anthropometric indicators. **Methods**: This prospective cross-sectional study included 58 children and adolescents aged 5–18 years with obesity and 30 normal-weight controls evaluated in Sfânta Maria Emergency Children’s Hospital Iași, Romania. Clinical data included anthropometric measurements and available biochemical parameters. RMR was assessed through indirect calorimetry (Fitmate Pro, Cosmed, Rome, Italy). Parents completed a structured lifestyle questionnaire adapted from validated international instruments, collecting information on physical activity, sedentary behavior, and wearable device use. Data analysis was conducted using SPSS 22.0, applying descriptive statistics and Pearson correlation analysis. **Results**: Children with obesity reported higher body mass index (BMI) (30.48 ± 5.31 kg/m^2^), higher RMR values, lower physical activity levels and greater sedentary time than controls. RMR correlated positively with BMI, central adiposity, blood pressure, waist-to-height, hepatic steatosis and exercise tolerance. Although electronic devices for monitoring physical activity were more frequently used in the obesity group, this was not associated with higher activity levels. **Conclusions**: Children and adolescents with obesity exhibited a clustered cardiometabolic risk profile and reduced physical activity. RMR measured by indirect calorimetry may contribute to a more comprehensive metabolic assessment in pediatric obesity.

## 1. Introduction

Childhood obesity remains one of the most challenging health problems worldwide in the 21st century and an important determinant of the early onset of cardiometabolic complications [1,2,3]. Recent global evidence indicates that the prevalence of obesity among children and adolescents was estimated at 8.5%, with evidence of an upward trend in more recent years and associated comorbidity risks such as hypertension and depression [4]. According to the sixth round (2022–2024) of the European Childhood Obesity Surveillance Initiative (COSI), which collected standardized anthropometric data across multiple European countries, approximately 28% of Romanian children aged 7–9 years were classified as being overweight and 12% as having obesity [5,6].

Excess adiposity in childhood and adolescence arises from a complex interaction of behavioral, biological, and environmental factors [7,8]. Unhealthy dietary patterns, low levels of physical activity, and sedentary behavior are among the main modifiable dysfunction-associated steatotic liver disease and increased long-term cardiovascular risk [9,10,11,12]. Because these adverse outcomes develop within the broader framework of energy imbalance, pediatric obesity should be evaluated not only by anthropometric measures but also by variables that reflect the physiological and behavioral aspects of energy expenditure [13,14]. In this context, resting metabolic rate (RMR) represents a major component of daily energy expenditure, whereas physical activity reflects a modifiable lifestyle-related determinant of metabolic health [15]. Their combined assessment may therefore offer a broader perspective on obesity-related cardiometabolic risk in children and adolescents than anthropometric indicators alone.

A rigorous evaluation of RMR can be central in understanding the metabolic rate and the metabolic profile of individuals with obesity. Indirect calorimetry enables the direct quantification of oxygen consumption and carbon dioxide production, offering an objective measurement of RMR rather than an estimation derived from population-based formulas [16]. Variability in RMR between individuals with similar anthropometric characteristics underscores the limitations of relying solely on predictive equations or body mass when estimating metabolic burden. Compared with predictive equations, indirect calorimetry offers an objective assessment of RMR. This is relevant in pediatric obesity, as commonly used equations may show limited accuracy and may not adequately reflect interindividual differences in body composition and metabolic characteristics [17,18,19]. However, its application in routine pediatric settings may remain constrained by equipment availability and logistical considerations.

Total energy expenditure also depends on physical activity-related energy consumption, which is the most variable component and a key determinant of metabolic health [20,21]. An altered RMR may signal adaptive metabolic responses, reduced metabolic flexibility, or early metabolic dysregulation, all of which can be linked to insulin resistance, adverse lipid profiles, and increased cardiometabolic risk [22,23].

Physical activity and RMR reflect complementary behavioral and physiological dimensions of energy expenditure in pediatric obesity. Physical activity is a modifiable behavior linked to cardiometabolic health, whereas RMR constitutes a major component of total energy expenditure and may add physiological context beyond anthropometric measures alone [24,25]. Their combined assessment may therefore contribute to a more comprehensive characterization of obesity-related cardiometabolic risk in children and adolescents. However, insufficient physical activity remains highly prevalent in youth. The World Health Organization has reported that a large majority of adolescents do not meet recommended activity levels, and physical inactivity combined with sedentary behavior contributes to noncommunicable disease risk across the life course [26,27]. Beyond energy balance, physical activity is clinically relevant because it is strongly linked to cardiorespiratory fitness, metabolic health, and blood pressure, and it may improve health outcomes even when weight loss is modest. At the same time, high sedentary time, particularly screen-based behaviors, is associated with higher body mass index (BMI) and obesity risk in adolescents [28]. Physical activity in pediatric populations can be assessed using either questionnaire-based approaches or objective monitoring tools. In the present study, physical activity was evaluated using a structured, validated questionnaire, which allowed for the characterization of habitual activity patterns, participation in organized sports, and sedentary behavior [29]. In pediatric populations, an average of at least 60 min per day of moderate-to-vigorous physical activity, mostly aerobic, together with vigorous and muscle- and bone-strengthening activities on at least 3 days per week, is recommended for metabolic and overall health and has been linked to more favorable body composition and cardiometabolic profiles [30,31,32,33]. Such a framework supports more precise and individualized preventive and therapeutic strategies in patients with overweight or obesity.

Although increasing physical activity is central to obesity management, sustained improvements remain difficult to achieve and maintain over time [34,35]. While physical activity and sedentary behavior have been widely studied in relation to pediatric obesity and cardiometabolic health, fewer studies have integrated these behavioral dimensions with measured RMR and metabolic characterization in clinical pediatric cohorts [14,36]. A tertiary clinical setting may facilitate the standardized assessment of pediatric obesity and its associated comorbidities [37]. Within this context, indirect calorimetry-based RMR measurement may add complementary physiological information to anthropometric and metabolic evaluation when available [38,39]. The tertiary care setting was not intended to imply routine RMR-based screening but rather provided the practical and clinical context in which objective metabolic assessment could be performed alongside the standardized evaluation of obesity-related comorbidities.

The primary aim of this cross-sectional study was to compare questionnaire-assessed physical activity patterns, sedentary behavior, RMR, and cardiometabolic characteristics between Romanian children and adolescents aged 5–18 years with obesity and normal-weight peers evaluated in a tertiary pediatric center. A secondary aim was to explore the associations between these variables within the study cohort. We hypothesized that children and adolescents with obesity would show lower physical activity, higher sedentary behavior, and a less favorable cardiometabolic profile than normal-weight peers.

## 2. Materials and Methods

### 2.1. Study Design and Participants

**Figure 1:**
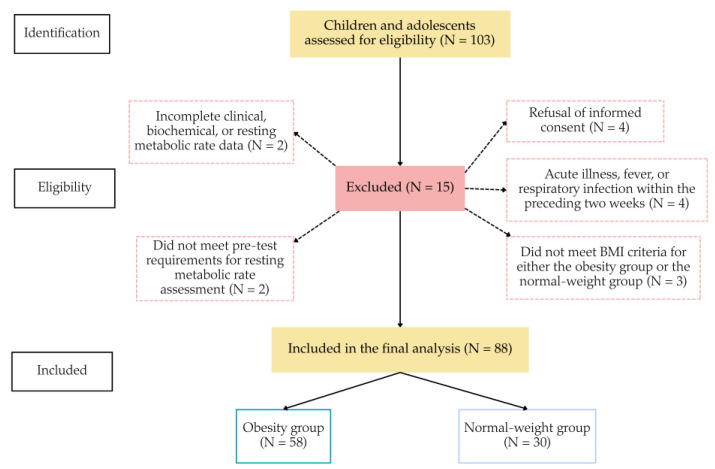
Flow diagram of participant recruitment, eligibility assessment, exclusions, and final inclusion in obesity and normal-weight groups.

This observational prospective cross-sectional clinical cohort study was conducted in a tertiary pediatric hospital between February and November 2025. Children and adolescents aged 5–18 years with a diagnosis of obesity, and their normal-weight peers, evaluated in Sfânta Maria Emergency Children’s Hospital in Iași, a city in the north-east of Romania, were eligible for inclusion. Obesity was defined according to age- and gender-specific BMI criteria based on World Health Organization growth references (BMI-for-age Z-scores > +2 standard deviation (SD)) [40]. The study protocol was approved by the institutional ethics committee and complied with the Declaration of Helsinki, and written informed consent was obtained from parents or legal guardians before participation. The participant recruitment and selection process is detailed in Figure 1, including the number of children and adolescents screened, excluded according to predefined criteria, and included in the final analysis.

Inclusion criteria:

- Patients aged 5–18 years at enrollment;
- Patients with obesity defined according to age- and gender-specific BMI using a single, pre-specified standard (World Health Organization growth reference for children—BMI-for-age > +2 SD), as well as matched participants with normal weight (BMI-for-age Z-scores between −1 and +1 SD);
- Participants with written informed consent obtained from a parent or legal guardian before the initiation of any study-related procedure;
- Patients who indicate adherence to standardized pre-test conditions for RMR assessment, including: an overnight fast of at least 8 h, absence of moderate-to-vigorous physical activity during the 24 h preceding the evaluation, avoidance of caffeine or other stimulant-containing beverages for at least 12 h before testing and the ability to tolerate the canopy hood system and remain in quiet rest for 15 min in the supine position throughout the assessment period.

Exclusion criteria:

- Refusal or withdrawal of informed consent.
- Patients presenting with medical conditions that could significantly influence RMR, including: acute illness, fever, or respiratory infection within the preceding two weeks; uncontrolled endocrine or metabolic disorders such as decompensated diabetes, Cushing syndrome, or other conditions known to substantially alter energy expenditure, including severe anemia or active malignancy; and severe psychiatric or neurodevelopmental disorders limiting the ability to cooperate with study procedures.
- Patients receiving chronic pharmacological treatments that may affect metabolic rate, including systemic glucocorticoids, chemotherapy, sympathomimetic agents, or other medications with metabolic effects.
- Known or suspected pregnancy in postmenarchal female participants.

### 2.2. Ethical Approval

The study protocol was reviewed and approved by the Ethics Committee of Saint Maria Emergency Children’s Hospital, Iasi, Romania (approval date 26 October 2023), and subsequently by the Research and Scientific Ethics Committee of Grigore T. Popa University of Medicine and Farmacy Iasi, Romania (approval no. 412; approval date: 11 March 2024). Written informed was obtained from the parents or legal guardians of all participants.

### 2.3. Data Collection

Children and adolescents aged 5 to 18 years were recruited and evaluated between February 2025 and November 2025. Clinical data were obtained from medical records and included: weight, height, BMI, waist circumference, biochemical parameters, abdominal ultrasonography, and information from nutritional consultations. Weight and height were measured using standardized procedures, with participants wearing light clothing and no shoes. BMI-for-age percentiles and Z-score were calculated using the World Health Organization (WHO) Anthro and WHO AnthroPlus software (version 1.0.4; World Health Organization, Geneva, Switzerland), based on the 2007 WHO growth reference standards [41].

Systolic and diastolic blood pressure (BP) were measured using a calibrated manual sphygmomanometer following standardized procedures. BP values were interpreted in accordance with the 2017 American Academy of Pediatrics guideline, using age-, sex-, and height-specific reference standards for children younger than 13 years and absolute blood pressure thresholds for adolescents aged 13 years and older [42,43,44]. Percentile values for systolic and diastolic BP were calculated using the Blood Pressure Percentiles, 0 to 17 years (2017 standard) clinical calculator available in the MSD Manuals Professional Edition (Merck & Co., Inc., Rahway, NJ, USA). For children younger than 13 years, age-, sex-, and height-specific percentiles tables were used, with normal BP defined as <90th percentile, elevated BP as ≥90th to <95th percentile, stage 1 hypertension as ≥95th percentile to <95th percentile + 12 mmHg, and stage 2 hypertension as ≥95th percentile + 12 mmHg. For adolescents aged 13 years and older, adult-like absolute thresholds were applied: normal BP < 120/<80 mmHg, elevated >120 to 129/<80 mmHg, stage 1 hypertension 130/80 to 139/89 mmHg, and stage 2 hypertension ≥140/90 mmHg [42,43,44].

Fasting venous blood samples were collected from all participants during the study period, after an overnight fast, at the time of clinical evaluation. The analyzed biochemical parameters included markers of glucose metabolism, the lipid profile, and inflammation. These parameters were investigated because they are part of the institutional diagnostic protocol routinely used in our hospital for the evaluation of pediatric obesity. All samples were analyzed in the hospital laboratory using routine standardized laboratory methods. These variables provide clinically relevant information for the assessment of obesity-related cardiometabolic risk and contribute to the overall clinical evaluation of children and adolescents with obesity [11].

RMR was measured by indirect calorimetry using a portable metabolic system (Fitmate software version 2.4 (build 29; COSMED S.R.L, Rome, Italy)) equipped with a ventilated canopy hood, according to the manufacturer’s protocol and under standardized resting conditions. This method was used as the available objective method for metabolic assessment in our hospital setting. Indirect calorimetry is considered the reference method for resting energy expenditure assessment [45,46]. However, device-specific validation data for the Fitmate Pro in children with obesity remain limited and should be considered when interpreting the results.

All assessments were performed in the morning under standardized laboratory conditions, and participants were instructed to fast overnight for at least 8 h and to avoid vigorous physical activity, caffeine, and other stimulant beverages prior to testing. Measurements were conducted in a thermoneutral, quiet environment. Participants rested in the supine position for 15 min for total recording time, with the first 5 min discarded to allow for stabilization. They were instructed to remain still, avoid speaking, and stay awake throughout the procedure. A 10 min steady-state period was selected for analysis based on commonly used indirect calorimetry criteria, namely <10% variability in oxygen consumption (VO_2_) and carbon dioxide (VCO_2_) and <5% variability in respiratory quotient, thresholds intended to ensure metabolic stability during measurement [47,48,49]. The Fitmate Pro device automatically calculated RMR using the abbreviated Weir equation:

RMR = [3.9 (VO_2_) + 1.1 (VCO_2_)] × 1.44

The Fitmate Pro system was prepared and calibrated in accordance with the manufacturer’s recommendations before each testing session, including flowmeter calibration using a 3 L syringe and standard internal sensor checks. Measurements were performed once per participant and under standardized pre-test conditions. This included overnight fasting for at least 8 h, the avoidance of moderate-to-vigorous physical activity during the previous 24 h and abstinence from stimulant-containing beverages for at least 12 h, followed by quiet supine rest before indirect calorimetry measurement. RMR values were interpreted according to the age-adjusted classification automatically generated by the calorimeter software using its built-in reference thresholds.

Body composition was assessed during a single clinical evaluation session using skinfold thickness measurements entered into Fitmate Pro software (Fitmate software version 2.4 (build 29; COSMED S.R.L, Rome, Italy)), which automatically estimated fat percentage, fat mass, and lean body mass using device-specific proprietary algorithms. A device-specific three-site protocol was applied according to the manufacturer’s body composition instructions, including upper thoracic measurements in boys, upper arm measurements in girls, and abdominal and thigh measurements in all participants and estimations using the Slaughter equation [50,51]. This approach provided a practical and standardized estimate of fat percentage, fat mass, and lean body mass in our clinical setting. However, because the device uses proprietary algorithms, the exact computational model is not fully transparent, which may limit external comparability with published prediction equations. Each skinfold was measured three times at the same anatomical site, and the mean value was used for analysis. Waist and hip circumferences were measured using a flexible, non-stretch anthropometric tape and subsequently entered into the system for the automatic calculation of the waist-to-hip ratio. Waist circumference was obtained in the standing position at the level of the lowest rib, with the tape placed horizontally and without skin compression. Hip circumference was measured in the standing position at the level of the maximum gluteal protuberance [52]. All anthropometric and calorimetric measurements were performed by the same two trained investigators, following standardized operating procedures throughout this study.

All participants underwent abdominal ultrasonography for the assessment of hepatic steatosis. The presence of fatty liver was recorded based on the ultrasound examination. The questionnaire used in this study was adapted from the International Study of Childhood Obesity, Lifestyle and the Environment (ISCOLE); the Child Feeding Questionnaire; and the WHO Childhood Obesity Surveillance Initiative instruments, using a previously validated Romanian version with demonstrated internal consistency and construct validity [29]. Parents or legal guardians completed a structured electronic questionnaire adapted from previously validated instruments.

The questionnaire collected information on:

- Demographic characteristics (urban/rural), educational level and socioeconomic status;
- Physical activity (frequency of moderate-to-vigorous activity, participation in organized sports, physical education classes);
- Sedentary behavior and screen time;
- Availability and use of wearable devices (type of device, frequency of use).

Physical activity and sedentary behavior were assessed using a structured electronic questionnaire completed by parents or legal guardians. This approach was selected to provide a consistent assessment method across the full study age range (5 to 18 years), particularly because younger children may have limited ability to accurately recall habitual physical activity. The questionnaire was adapted from previously validated instruments and was used to capture weekly moderate-to-vigorous physical activity, participation in organized sports, physical education attendance, sedentary behavior, and screen time in a feasible manner within routine clinical assessment [29]. Physical activity was assessed as the number of days per week with at least 60 min of moderate-to-vigorous physical activity, based on international recommendations for children and adolescents [33]. For analytical purposes, questionnaire responses were grouped as having low (<3 days/week), intermediate (3–4 days/week), or higher (>5 days/week) weekly activity frequency. This categorization was intended as a pragmatic interpretation of questionnaire-derived data rather than as a universally validated severity classification. A child-completed self-report instrument was not used as the sole assessment method because the cohort included younger children, and the accuracy of self-reported habitual physical activity is more limited in lower age groups [53]. A proxy-based format was therefore chosen to maintain a uniform method across the entire cohort. Information on wearable devices was collected only descriptively and referred only to device availability, type of device, and reported frequency of use. Objective monitoring with wearables or accelerometry was not used for physical activity quantification in the present study. Although device-based methods provide more objective movement estimates, they were not incorporated into the study design because the assessment was performed under routine hospital conditions and relied on standardized questionnaire-based and clinical measurements. In addition, device-based methods are more resource-intensive and do not provide the same information on the type and context of activity behavior [54,55]. Because no standardized wearable-derived activity data were recorded, questionnaire-based physical activity estimates could not be validated against device measurements in the present study.

### 2.4. Statistical Analysis

Statistical analyses were performed using SPSS version 22.0 (IBM Corp., Armonk, New York, NY, USA). Descriptive statistics were calculated for all variables. Continuous data were expressed as the mean ± standard deviation for normally distributed variables or as the median and interquartile range (IQR) when distributional assumptions were not met. Categorical variables were presented as percentages. Distribution normality was assessed using graphical methods, including frequency histograms.

Associations between physical activity parameters, RMR, and biochemical parameters were evaluated using correlation analysis, with Spearman correlation coefficients applied for non-normally distributed variables. Statistical analyses were performed using a 95% confidence level and a significance threshold of *p* < 0.05. A formal a priori sample size calculation was not performed, and the final sample consisted of all eligible participants recruited during the predefined study period according to the inclusion and exclusion criteria.

Comparisons between two independent groups were performed using the independent samples t test for normally distributed continuous variables and the Mann–Whitney U test for variables with non-normal distribution; the results are presented as the mean ± SD or median (IQR), with corresponding *p*-values and 95% confidence intervals where applicable. All statistical tests were two-sided, and a *p*-value <0.05 was considered statistically significant.

## 3. Results

### 3.1. Participant Characteristics

The study cohort consisted of 58 children and adolescents with obesity and 30 normal-weight controls. The mean age was similar between groups (11.91 ± 3.31 years vs. 12.77 ± 3.319 years; 95% confidence interval (CI) −2.33 to 0.63). The descriptive characteristics of the study cohort, including sex distribution and environment of origin, are presented in the Supplementary Materials (Figure S1, Tables S1 and S2).

### 3.2. Anthropometric and Body Composition Characteristics

**Figure 2:**
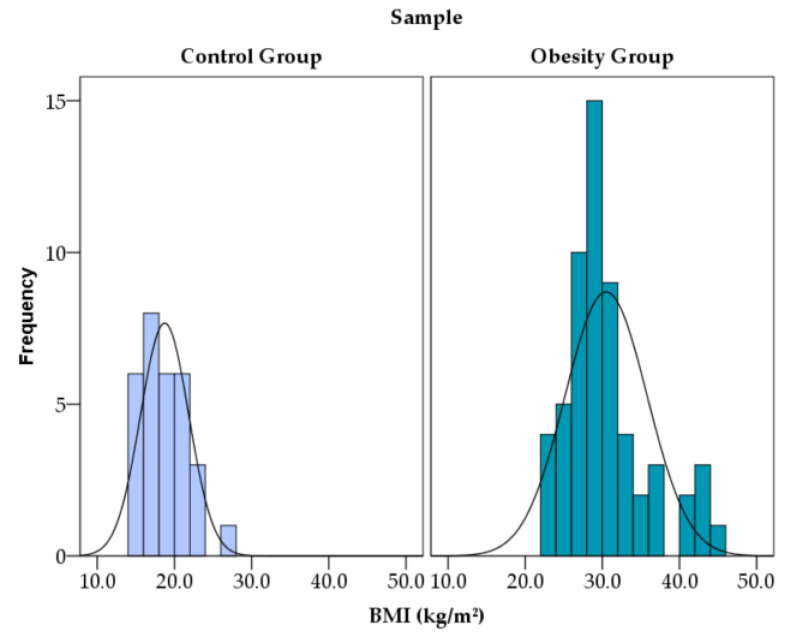
Distribution of BMI in study groups.

As expected in the comparison of the study cohort, significant differences were observed in anthropometric parameters. Compared with controls, participants with obesity had markedly higher body weight (95% CI 21.69 to 39.89), BMI (95% CI 9.65 to 13.85) (Figure 2), and BMI z-score, while the mean height was similar between groups.

Central adiposity indicators were consistently higher in participants with obesity. The mean waist circumference was substantially higher in the obesity group than in controls (90.74 ± 14.54 cm vs. 63.63 ± 8.85 cm), and body composition analysis showed both a higher fat mass percentage and a markedly greater total fat mass.

In the obesity group, 51 out of 58 children had a waist circumference above the 90th percentile, interpreted according to the International Diabetes Federation criteria for pediatric metabolic syndrome [56,57]. In contrast, none of the participants in the normal-weight group had a waist circumference exceeding the 90th percentile. The waist-to-hip ratio was slightly higher in participants with obesity compared with the control group. In the normal-weight group, the mean waist-to-hip ratio was 0.90 ± 0.06, with values ranging from 0.64 to 1.00. In the obesity group, the mean ratio was 0.91 ± 0.05, with a range of 0.76 ± 1.08. Although the absolute difference in mean values between groups was modest, the obesity group demonstrated a narrower distribution and higher minimum values, suggesting a consistently greater degree of central fat distribution compared with normal-weight peers.

The mean waist-to-height ratio was 0.582 ± 0.063 in the obesity group and 0.414 ± 0.034 in the control group. Based on the threshold of 0.50 for central adiposity, the majority of children with obesity met the criteria for increased cardiometabolic risk, whereas none of the normal-weight participants reached this cutoff.

### 3.3. Resting Metabolic Rate

**Figure 3:**
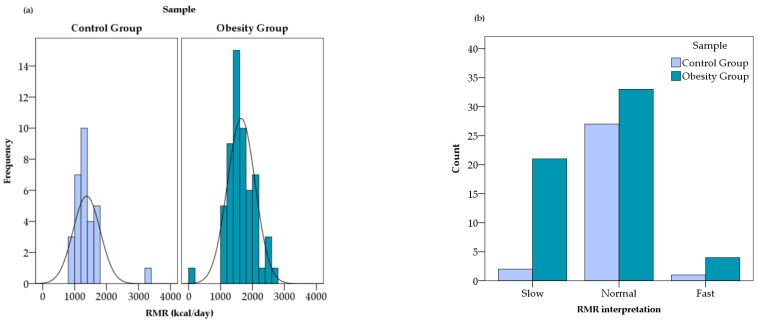
Distribution of RMR (**a**) and its interpretation (**b**) in study cohort.

Resting metabolic rate was elevated in the obesity group (1648.67 ± 435.55 kcal/day) compared with controls (1375 ± 425.07 kcal/day), consistent with increased absolute body mass and lean mass (Figure 3a). Based on the RMR values obtained by indirect calorimetry, the device software automatically classified participants according to the age-adjusted metabolic rate thresholds in Figure 3b. According to the device thresholds, normal metabolic range predominated in both groups, although slow RMR classification was more frequent in the obesity group than in controls. Fast metabolic rate classification remained uncommon in both groups.

### 3.4. Clinical and Metabolic Characteristics

BP values were consistently higher in the obesity group compared with the control group. In the control group, the mean systolic BP was 106.30 ± 6.83 mmHg, while the mean diastolic BP was 69.13 ± 8.57 mmHg. In contrast, children and adolescents with obesity had a mean systolic BP of 118.29 ± 14.06 mmHg and a mean diastolic BP of 80.36 ± 9.01 mmHg. The dispersion of systolic values was notably greater in the obesity group, as reflected by the higher standard deviation and a wider range. A similar pattern was observed for diastolic BP, with higher absolute values and greater variability in the obesity group compared with controls.

**Figure 4:**
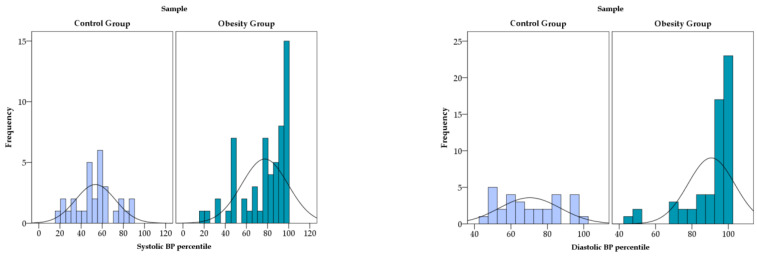
Distribution of systolic and diastolic blood pressure percentiles in study group.

The frequency distributions of systolic and diastolic BP percentiles are presented in Figure 4. As illustrated, the control group shows a relatively symmetric distribution centered within normal percentile ranges. In comparison, the obesity group demonstrates a rightward shift in both systolic and diastolic percentile distributions, with a higher concentration of participants in the upper percentile categories. This pattern indicated a greater burden of elevated BP values among children and adolescents with obesity.

A comprehensive biochemical and hematological panel was evaluated in all study participants (Table S3 in Supplementary Materials). Analyzing metabolic and glycemic parameters, children and adolescents with obesity exhibited clear evidence of insulin resistance. In the study group, participants with obesity showed higher insulin levels (42.01 ± 15.81 μIU/mL vs. 34.71 ± 22.98 μIU/mL), higher glycated hemoglobin A1C (HbA1c), and higher HOMA index values than controls, whereas fasting glucose remained within the normal range in both groups. These findings indicate early disturbances in glucose homeostasis, primarily driven by hyperinsulinemia.

The obesity group showed a more atherogenic lipid profile than controls, with higher total cholesterol, LDL cholesterol, and triglyceride levels, together with lower HDL cholesterol. In addition, 11 participants in the obesity group had triglyceride levels ≥150 mg/dL.

As an inflammatory marker, C-reactive protein values were elevated in both groups but showed greater variability in children with obesity. The erythrocyte sedimentation rate was also higher in the obesity group (11.50 ± 8.28 mm/h versus 7.80 ± 10.89 mm/h), supporting the presence of low-grade inflammation.

Alanine aminotransferase (ALT) levels were substantially higher in the obesity group (30.21 ± 18.08 U/L versus 18.93 ± 10.46 U/L), showing the hepatic involvement consistent with metabolic-associated steatotic liver disease. Given the presence of ultrasonographic-confirmed hepatic steatosis in 34 patients in the obesity group, these biochemical findings reinforce the hepatic component of cardiometabolic risk.

Uric acid levels were elevated in the obesity group (5.85 ± 1.10 mg/dL versus 4.20 ± 0.60 mg/dL). Creatinine and urea values remained within expected pediatric reference ranges (Table 1).

Baseline demographic characteristics were comparable between groups, with no significant differences between groups in age or height. Children and adolescents with obesity demonstrated a consistent pattern of excess total and central adiposity, reflected by significantly higher BMI, Z-score, and circumferential measures (all *p* < 0.001). Fat mass and lean body mass were both significantly increased, accompanied by a higher RMR (*p* = 0.006).

Correlation analyses identified multiple statistically significant associations between adiposity measures, metabolic parameters, and cardiometabolic risk indicators. The complete correlation matrix is presented in Table 2, while only the associations most important to the study objectives are described.

RMR showed a moderate positive correlation with BMI and stronger associations with markers of central adiposity, including hip and waist circumferences (all *p* < 0.01). A significant positive association was observed between RMR and lean body mass (r = 0.78, *p* < 0.01).

Anthropometric indices were strongly intercorrelated. BMI demonstrated very strong associations with abdominal, hip, and waist circumferences (r > 0.93, all *p* < 0.01), as well as with fat mass. The waist-to-height ratio showed significant positive correlations with anthropometric parameters, BP values, and the HOMA index (all *p* < 0.01).

BP values were positively associated with measures of adiposity. Both systolic and diastolic BP showed moderate correlations with BMI and circumferences (r values between 0.53 and 0.68, all *p* < 0.01).

Regarding metabolic markers, triglyceride concentrations were positively associated with adiposity measures and the HOMA index, whereas HDL cholesterol showed inverse correlations with BMI, waist circumference, and triglycerides, all with *p* < 0.01, consistent with an atherogenic lipid profile.

The markers of glucose metabolism showed similar trends. Insulin levels and the HOMA index demonstrated positive correlations with the waist-to-height ratio, triglycerides, and hepatic steatosis (all *p* < 0.01), suggesting early insulin resistance in participants with greater adiposity. Hepatic steatosis was significantly associated with BMI (r = 0.50, *p* < 0.01), waist circumference, RMR (r = 0.33, *p* < 0.01), and triglycerides, reinforcing the clustering of metabolic alterations associated with obesity.

Exercise tolerance was inversely associated with RMR (r = −0.30, *p* < 0.01), BMI (r = −0.70, *p* < 0.01), z-score, waist circumference, systolic and diastolic BP, waist-to-height, triglyceride levels, and hepatic steatosis and showed positive associations with HDL cholesterol, indicating that lower physical fitness is linked to a less favorable cardiometabolic profile.

### 3.5. Social and Lifestyle-Related Characteristics

Table 3 summarizes the sociodemographic profile, physical activity-related indicators, and selected lifestyle characteristics of the study participants, including educational level, socioeconomic status, weekly training volume, and daily screen time.

Children’s educational level differed modestly between groups, with control participants more frequently represented in higher educational categories, whereas the obesity group was more often concentrated in earlier educational stages (Table 3). Parent-reported socioeconomic status showed only modest variation between-group, with middle status predominating in both groups, although low and high categories were relatively more frequent in the obesity group.

**Figure 5:**
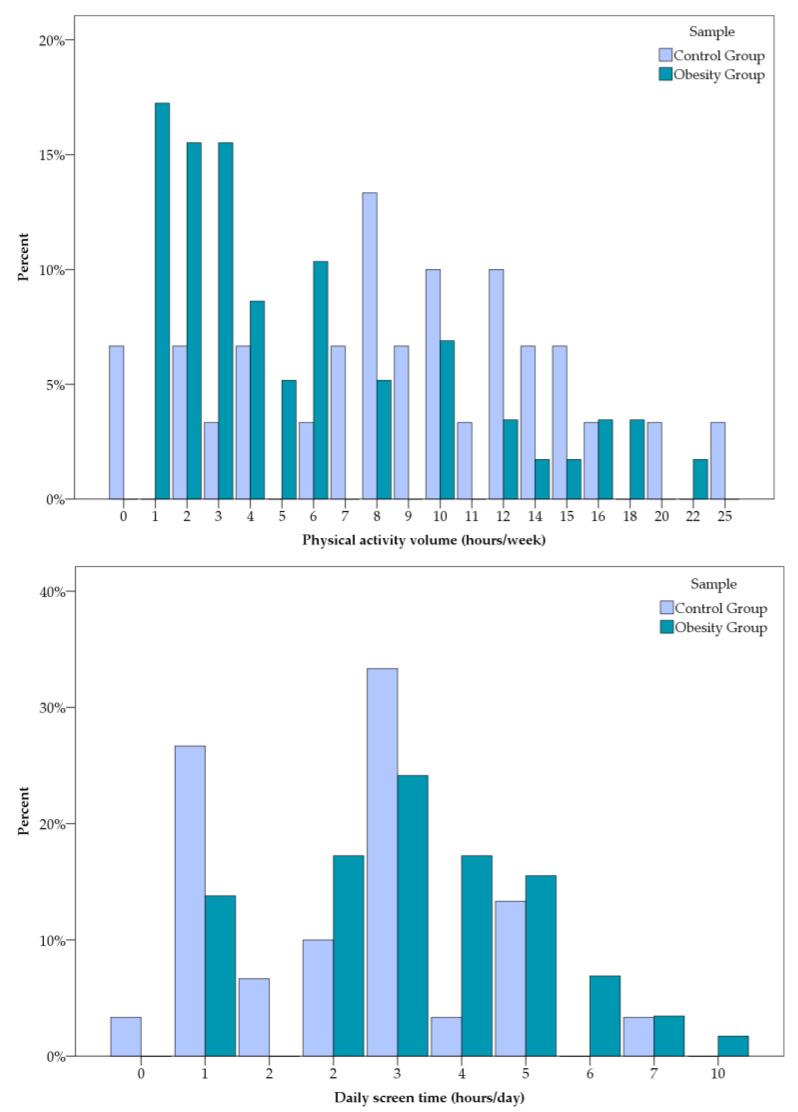
Graphical representation of physical activity patterns and sedentary behavior.

Differences in reported weekly physical activity volume, sedentary behavior, and participation in organized sports were observed between the obesity and control groups (Figure 5). Compared with controls, participants with obesity were more frequently classified in the lower weekly physical activity categories and reported less participation in organized sports, while controls showed a broader spread of activity levels and greater engagement in structured physical activity.

Sedentary behavior, assessed through daily screen time (Figure 5), also differed between groups. In the control group, screen time values were more evenly distributed and generally clustered between one to three hours per day, with only a small proportion reporting prolonged screen exposure. In the obesity group, daily screen time was shifted toward higher values. Most participants reported between two to five hours of screen use per day, with several individuals exceeding six hours daily.

**Figure 6:**
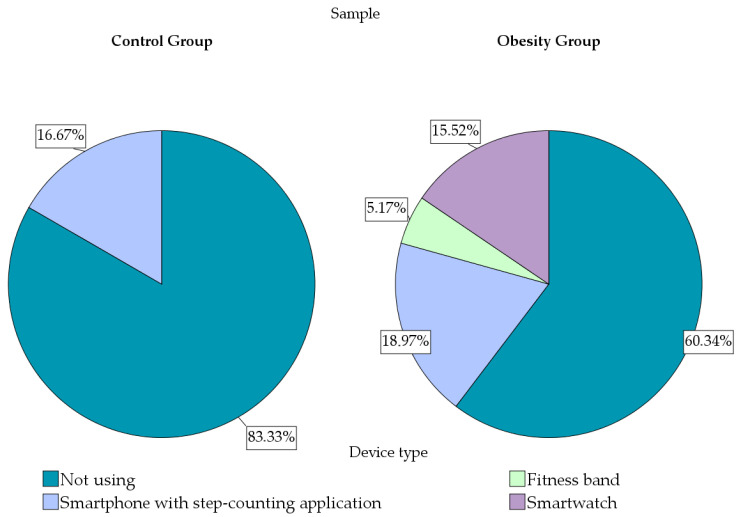
Use of electronic devices for monitoring physical activity.

The use of activity-monitoring devices remained limited in both groups, although it was reported more frequently in participants with obesity than in normal-weight controls (Figure 6). Overall, most participants in both groups did not use such devices, while smartphone applications and wearable devices were more commonly reported in the obesity group.

## 4. Discussion

Childhood and adolescence are critical developmental periods characterized by increased vulnerability to obesity and its associated health complications, commonly driven by unhealthy dietary patterns and sedentary behavior [58,59]. The present study investigated the relationship between physical activity patterns, RMR, and cardiometabolic risk factors in children and adolescents with obesity. In this cross-sectional study, Romanian children and adolescents with obesity differed from their normal-weight peers in questionnaire-assessed physical activity, sedentary behavior, RMR, and cardiometabolic characteristics. Compared with controls, participants with obesity showed lower engagement in organized physical activity and less favorable clinical and metabolic findings. These results represent descriptive and comparative observations within the study cohort and should be interpreted accordingly.

A recent position paper also emphasizes the systematic assessment of hypertension, dyslipidemia, impaired glucose metabolism, and metabolic dysfunction-associated steatotic liver disease in pediatric obesity, highlighting cardiometabolic risk as a routine care target rather than an exception [12]. The present findings directly support this approach: although fasting glycemic indices were not different, the cardiovascular and lipid signals were already abnormal in the obesity group.

Our cohort shows a high burden of adiposity and central fat distribution, accompanied by adverse functional and metabolic profiles. These findings are consistent with evidence from the literature indicating that abdominal adiposity represents one of the strongest predictors of early cardiometabolic risk in pediatric populations [12]. Central fat accumulation is closely associated with metabolic dysfunction, including dyslipidemia, insulin resistance, and elevated BP, which may contribute to the early development of cardiovascular disease [60,61,62]. Also, the waist-to-height ratio was a reliable screening tool for identifying children at risk for cardiometabolic disorders [63]. A large multinational analysis (24,605 children for derivation plus 9619 for external validation across multiple countries) showed that a single universal pediatric waist-to-height ratio cutoff is not uniformly optimal across regions, but the waist-to-height ratio remains a useful screen for identifying children with multiple cardiometabolic risk factors [64]. In our cohort, the higher waist-to-height ratio observed in the obesity group aligns with this evidence and provides a defensible rationale for including the waist-to-height ratio in routine clinical assessment alongside BMI and the BMI z-score.

Consistent with previous research, children with obesity in our cohort also exhibited unfavorable lipid profiles, including higher LDL cholesterol and triglyceride levels and lower HDL cholesterol concentrations. Correlation analyses indicated that RMR tracked positively with lean body mass and adiposity (all *p* < 0.01), while adiposity markers correlated with blood pressure, lipids, insulin resistance indices, and hepatic steatosis, reinforcing central adiposity as a clinically meaningful risk signal. These findings align with recent pediatric evidence and current guidance emphasizing early cardiometabolic screening, increased structured physical activity, and caution against assuming that wearable monitoring alone changes behavior [11,33,64]. Childhood obesity is widely recognized as a major driver of early metabolic disturbances, and importantly, these metabolic changes may persist into adulthood and contribute to long-term cardiovascular disease risk [12].

The findings in the study’s analysis suggest that children and adolescents with obesity were more likely to use digital tools for physical activity monitoring than their normal-weight peers. However, despite the higher adoption of wearable or smartphone-based monitoring devices in the obesity group, this did not appear to translate into higher levels of physical activity, as the same group reported lower participation in organized sports and lower weekly training volumes.

The metabolic abnormalities observed in our cohort may also be interpreted within a broader pathophysiological framework. Skeletal muscle is the major site of insulin-mediated glucose disposal and accounts for a large proportion of postprandial glucose intake [30,31,65,66]. This means that reduced physical activity may contribute to impaired glucose handling, whereas exercise may improve insulin sensitivity by enhancing skeletal muscle glucose uptake and metabolic flexibility [67]. This mechanism is relevant in the context of our findings, as children and adolescents with obesity showed higher insulin levels, elevated HOMA index values, and higher HbA1c levels, supporting the presence of early disturbances in glucose homeostasis. In addition, obesity has been linked in the literature to adipokine imbalance and mitochondrial dysfunction, which may further impair insulin signaling and metabolic flexibility [68,69,70,71,72,73]. However, these mechanisms were not directly assessed in the present study and should therefore be interpreted as biologically plausible explanatory pathways rather than direct findings.

From a cardiometabolic perspective, obesity is associated with chronic low-grade inflammation and oxidative stress, both of which may contribute to metabolic dysfunction [68,74]. Regular physical activity may help attenuate oxidative stress through the adaptative upregulation of antioxidant defenses and improvement in mitochondrial efficiency [74]. In our cohort, children and adolescents with obesity had a more atherogenic lipid profile, with higher total cholesterol, LDL cholesterol, and triglycerides, together with lower HDL cholesterol, as well as higher inflammatory markers. These findings support the presence of early cardiometabolic impairment rather than isolated excess body weight alone. Current evidence indicates that physical activity in pediatric obesity is associated with better insulin sensitivity and may contribute to a more favorable cardiometabolic profile [12,75,76,77]. Studies in pediatric populations have shown that obesity may already be associated with increased carotid intima–media thickness and altered vascular function, supporting the relevance of such assessments in future research [78,79]. Future studies should integrate lifestyle evaluation with cardiovascular assessment in order to better characterize the early cardiometabolic impact of pediatric obesity. Measuring RMR by indirect calorimetry can be a major methodological strength, because predictive equations can be inaccurate at the individual level. This method is widely regarded as the reference standard for measuring energy expenditure, because indirect calorimetry allows for a more precise assessment of individual energy metabolism [22]. Bedogni et al. externally validated 10 resting energy expenditure equations in 2426 children (2037 with obesity and 389 without) and found that even the best-performing equation had only moderate “correct classification” within 10% of measured values, especially in obesity [80]. This supports the framing of indirect calorimetry as a diagnostic-grade physiological measurement that can meaningfully refine energy prescription in research and, selectively, in clinical care.

The findings of the present study regarding RMR are consistent with previous research examining energy expenditure in individuals with obesity [22,81,82]. In our cohort, children with obesity presented significantly higher absolute RMR values compared with the control group, which can largely be explained by their greater body mass and higher lean body mass. Similar observations have been reported in the literature, where obese individuals typically demonstrate higher absolute energy expenditure compared with their non-obese counterparts. However, this difference is primarily driven by differences in body composition, particularly fat-free mass, which is the main determinant of RMR [13]. Carneiro et al. emphasized that obese individuals generally exhibit increased absolute energy expenditure because fat-free mass increases concomitantly with body weight, and metabolically active tissues contribute substantially to basal energy requirements [83]. Importantly, when energy expenditure is adjusted for body composition, especially fat-free mass, the apparent differences between obese and non-obese individuals often diminish, suggesting that obesity itself is not necessarily associated with an intrinsically “slow metabolism” [13,83]. Our results also support the concept that higher RMR in obesity does not reflect improved metabolic health but rather represents the energetic cost of maintaining a larger body mass. Absolute energy expenditure increases with body size, while behavioral factors such as reduced physical activity may contribute more substantially to the maintenance of obesity and cardiometabolic risk. Overall, the correlation analysis in our sample supports the presence of a tightly interconnected cardiometabolic network in which central adiposity appears to be the key driver, linking increased RMR, insulin resistance, dyslipidemia, elevated BP, and hepatic involvement in this pediatric population.

Lifestyle differences in the cohort were directionally consistent with a risk-amplifying environment: children with obesity reported less organized sports participation and more sedentary behavior. This behavioral pattern may contribute to the clustering of cardiometabolic risk factors observed in the obesity group.

Recent evidence supports the relevance of organized sport as an actionable lever. Danielsen et al. studied 85 youth with severe obesity matched to 85 normal-weight peers and found that less time was spent on moderate and vigorous activity in the obesity group; within the obesity group, participation in organized sports was associated with more moderate activity and greater vigorous activity [84]. This is a strong comparator because it links an accessible intervention target (organized sport) with objectively measured activity differences. The distribution of screen time indicates a greater prevalence of prolonged sedentary behavior among children and adolescents with obesity. Physical activity patterns also differed significantly between groups, and participants in the obesity group reported lower levels of participation in organized sports and a higher prevalence of sedentary behaviors. Reduced physical activity is a key modifiable factor contributing to cardiovascular risk in youth. A systematic review and meta-analysis including seven studies found a linear positive association between screen time and metabolic syndrome risk in children and adolescents, with higher odds of metabolic syndrome per incremental increase in daily screen time [85]. In the study group, higher sedentary behavior in obesity fits this literature and supports framing sedentary reduction as part of cardiometabolic risk mitigation, not merely weight management. Evidence indicates that regular physical activity can reduce visceral fat, improve insulin sensitivity, and favorably influence lipid profiles and BP in children and adolescents [86,87,88]. Therefore, insufficient engagement in physical activity may represent an important mechanism linking obesity with cardiometabolic risk in pediatric populations.

Interestingly, although the use of wearable devices and smartphone applications for monitoring physical activity was more frequent among children with obesity, this did not translate into higher physical activity levels. In adults, wearable-based interventions have demonstrated modest but measurable improvements in daily step count and cardiometabolic parameters, compared with usual care interventions [89,90]. Evidence in pediatric populations is growing but remains heterogeneous, with most studies conducted in school-based or experimental environments rather than routine clinical care [91]. In the present cohort, the reported use of digital monitoring devices was limited and did not appear to coincide with more favorable physical activity patterns at the descriptive level. However, no conclusions can be drawn regarding the effectiveness of digital self-monitoring, as this was not an interventional study. This is consistent with the concept that monitoring is not treatment. Au et al., in a 2024 systematic review and meta-analysis of 21 randomized trials (3676 participants), found that wearables led to increased daily steps but did not lead to significantly increased moderate-to-vigorous physical activity, suggesting that devices can influence a shift in low-intensity movement without reliably resulting in a change in higher-intensity behaviors [92]. Similarly, a 2022 meta-analysis of 12 randomized controlled trials (3227 participants) evaluating wearable interventions for pediatric obesity outcomes found modest benefits in terms of body mass index and body mass index z-score, with less consistent effects on waist circumference, reinforcing that wearables alone are unlikely to normalize central adiposity or cardiometabolic risk without structured behavioral support [93]. Instead, wearable technologies may be more effective when integrated into structured lifestyle interventions that include behavioral counseling and supervised exercise programs.

### Strengths and Limitations

This study integrates anthropometric, biochemical, and behavioral data to provide a comprehensive evaluation of cardiometabolic risk in pediatric obesity. In addition, the use of indirect calorimetry represents an important methodological strength in the evaluation of patients with obesity, allowing for an objective assessment of RMR. However, several limitations should be acknowledged. The cross-sectional design does not allow for causal inference regarding the relationship between physical activity patterns and cardiometabolic outcomes. Physical activity was assessed using a parent-reported questionnaire rather than objective device-based monitoring, without validation against wearable-derived data, which may introduce recall and social desirability bias and possible misclassification and limit measurement precision. Although indirect calorimetry provided an objective assessment of RMR, validation data specifically for the Fitmate Pro device in children with obesity remain limited. Portable indirect calorimetry was used as the objective method available in our hospital for RMR assessment under routine clinical conditions. This choice reflected feasibility in a pediatric outpatient setting rather than methodological superiority over whole-room calorimetry, which was not available. Although this device provided an objective assessment, it remains method-dependent and may be influenced by protocol standardization, steady-state assumptions, and device-specific precision limitations. In addition, the present study does not support conclusions regarding the role of RMR in screening, risk stratification, or routine obesity management. Future longitudinal studies using objective activity-monitoring methods and more comprehensive metabolic assessment are needed to further clarify the relationships between physical activity, energy metabolism, and cardiometabolic risk in pediatric populations.

A further limitation of this study is that ethnicity was not specifically recorded. Therefore, its potential influence on physical activity patterns, lifestyle behaviors, and obesity-related differences could not be assessed. Although all participants were recruited from Romania and from a relatively limited geographical area, which may have reduced ethnic heterogeneity, unmeasured ethnic variation cannot be entirely excluded. In addition, socioeconomic status was assessed only broadly, despite being reported in this study, and more detailed information such as parental education, occupation, and household income was not available. Dietary habits and sleep patterns were likewise not characterized in sufficient detail, limiting a more nuanced interpretation of lifestyle-related differences between study groups. Furthermore, stress and other psychological factors were not assessed, although they may meaningfully influence eating behavior and obesity-related outcomes. Finally, this study did not include detailed cardiological or vascular assessments, including markers of subclinical vascular remodeling such as carotid intima–media thickness, arterial stiffness, or endothelial function. While these measures were beyond the primary scope of the present work, they would have strengthened the cardiometabolic interpretation of the findings. Accordingly, the results should be interpreted within the context of these methodological constraints. Future studies should include detailed nutritional assessment, standardized socioeconomic indicators, sleep assessment, and psychological and behavioral variables such as perceived stress in order to provide a more comprehensive interpretation of lifestyle-related differences.

The questionnaire was completed by parents or legal guardians in order to ensure a uniform assessment approach across the fully study age range, including younger children for whom the self-reporting of habitual physical activity may be less reliable. This method also allowed for the collection of contextual information regarding activity type, organized sports participation, and sedentary behavior within routine conditions. However, proxy-reported questionnaires remain subject to recall and social desirability bias and do not provide the same level of precision as device-based objective monitoring. Objective wearable-derived activity measurement was not included in the study design, and information on wearable devices was collected descriptively only.

## 5. Conclusions

Children and adolescents with obesity exhibited a clustered cardiometabolic risk profile characterized by central adiposity, dyslipidemia, elevated blood pressure, and reduced participation in organized physical activity. Beyond excess adiposity, low physical fitness and sedentary behavior contribute substantially to long-term risk and reduced quality of life in affected children.

Central adiposity markers such as waist circumference and the waist-to-height ratio were strongly associated with metabolic and cardiovascular risk factors, reinforcing their value as screening indicators in pediatric clinical practice.

The resting metabolic rate measured by indirect calorimetry showed significant relationships with body composition parameters and provided valuable insight into individual energy metabolism. The integration of this method into clinical evaluation protocols may improve the diagnostic characterization of pediatric obesity and support personalized lifestyle interventions. The accurate measurement of RMR can improve the estimation of individual energy requirements and support the development of personalized dietary and physical activity interventions.

Overall, strategies aimed at increasing physical activity, reducing sedentary behavior, and improving metabolic assessment may play a key role in preventing and managing cardiometabolic risk in children and adolescents with obesity.

## Figures and Tables

**Table 1:** Comparison of clinical and metabolic characteristics between children with obesity and normal-weight controls.

	Obesity Group (*N* = 58)			Control Group (*N* = 30)				*T*-Test						
	Mean	±SD		Mean		±SD		t (df)		*p*		Mean Difference	95% CI	
													Lower	Upper
Age (years)	11.91	3.315		12.77		3.319		−1.14 (86)		0.25		−0.853	−2.336	0.630
Weight (kg)	76.626	26.7218		45.833		16.0824		6.73 (86)		<0.001		30.7925	21.6940	39.8910
Height (cm)	156.07	18.179		153.80		18.227		0.55 (86)		0.581		2.269	−5.866	10.403
BMI (kg/m^2^)	30.488	5.3189		18.733		3.1192		11.13 (86)		<0.001		11.7546	9.6562	13.8530
Z-score	3.2040	0.92636		−0.1160		0.83889		16.44 (86)		<0.001		3.31997	2.91859	3.72135
Abdominal circumference (cm)	96.12	15.499		65.50		9.755		9.84 (86)		<0.001		30.621	24.437	36.804
Hip circumference (cm)	99.69	15.029		71.10		10.420		9.31 (86)		<0.001		28.590	22.487	34.692
Waist circumference (cm)	90.74	14.542		63.63		8.857		9.33 (86)		<0.001		27.108	21.338	32.879
RMR (kcal/day)	1648.67	435.557		1375.00		425.074		2.81 (86)		0.006		273.672	80.519	466.826
FAT%	30.864	6.0271		15.927		6.4081		10.78 (86)		<0.001		14.9371	12.184	17.6902
Fat body mass (kg)	24.6655	11.42153		7.6870		3.92848		10.21 (86)		<0.001		16.97852	13.66894	20.28809
Lean body mass (kg)	51.3116	16.32286		38.6787		13.77095		3.62 (86)		<0.001		12.63289	5.69924	19.56653
Systolic BP (mmHg)	118.29	14.056		106.30		6.834		5.38 (86)		<0.001		11.993	7.564	16.422
Diastolic BP (mmHg)	80.36	9.014		69.13		8.573		5.63 (86)		<0.001		11.229	7.264	15.193
Waist-to-hip ratio	0.9117	0.05629		0.8997		0.06631		0.89 (86)		0.37		0.12	−0.0147	0.03882
Waist-to-height ratio	0.5819	0.06377		0.4142		0.03420		16.05 (86)		<0.001		0.16772	0.14695	0.18848
LDL-C (mg/dL)	101.07	26.41		82.04		17.09		4.07 (86)		<0.001		19.02747	9.74323	28.31172
Total cholesterol (mg/dL)	157.63	25.35		141.33		21.15		3.01 (86)		0.003		16.30460	5.56490	27.04429
Insulin (uIU/mL)	42.00	15.80		34.71		22.97		1.55 (86)		0.126		7.29663	−2.13917	16.73242
	Obesity Group (*N* = 58)						Control Group (N = 30)						Mann–Whitney U	
	Mean Rank		Median		IQR		Mean Rank		Median		IQR		**U**	***p***
Triglycerides (mg/dL)	29.18		70.0		23.25		52.42		99.50		58.00		410.50	<0.001
HDL-C (mg/dL)	54.97		51.50		13.50		39.09		48.00		11.50		556.00	0.006
Fasting glucose (mg/dL)	49.47		86.0		20.25		41.93		84.00		11.00		721.00	0.189
HOMA score	38.57		6.90		6.95		47.57		8.40		4.30		692.00	0.117

*N* = sample size; SD = standard deviation; df = degrees of freedom; CI = confidence interval; BMI = body mass index; RMR = resting metabolic rate; FAT% = body fat percentage; BP = blood pressure; LDL-C = low-density lipoprotein cholesterol; HDL-C = high-density lipoprotein cholesterol; IQR = interquartile range; HOMA = homeostatic model assessment of insulin resistance.

**Table 2:** Correlations between RMR and anthropometric data.

	1	2	3	4	5	6	7	8	9	10	11	12	13	14	15	16	17	18	19	20	21
Pearson Correlations																					
1. RMR (kcal/day)	1																				
2. BMI (kg/m^2^)	0.58 **	1																			
3. Z-score	0.30 **	0.81 **	1																		
4. Age (years)	0.46 **	0.24 *	−0.25 *	1																	
5. Abdominal circumference (cm)	0.66 **	0.93 **	0.73 **	0.32 **	1																
6. Hip circumference (cm)	0.65 **	0.94 **	0.67 **	0.42 **	0.94 *	1															
7. Waist circumference (cm)	0.69 **	0.94 **	0.69 **	0.36 **	0.98 **	0.94 **	1														
8. Systolic BP (mmHg)	0.53 **	0.63 **	0.45 **	0.35 **	0.68 **	0.65 **	0.68 **	1													
9. Diastolic BP (mmHg)	0.49 **	0.55 **	0.47 **	0.22 *	0.60 **	0.61 **	0.58 **	0.67 **	1												
10. Fat body mass (kg)	0.59 **	0.82 **	0.56 **	0.37 **	0.85 **	0.86 **	0.85 **	0.65 **	0.58 **	1											
11. Lean body mass (kg)	0.78 **	0.68 **	0.32 **	0.68 **	0.76 **	0.77 **	0.77 **	0.57 **	0.50 **	0.67 **	1										
12. Waist-to-hip ratio	0.18	0.09	0.17	−0.22 *	0.20	−0.06	0.24 *	0.16	−0.05	0.05	0.05	1									
13. Waist-to-height ratio	0.37 **	0.89 **	0.89 **	−0.10	0.84 **	0.76 **	0.84 **	0.51 **	0.45 **	0.66 **	0.40 **	0.34 **	1								
14. LDL-C (mg/dL)	0.04	0.26 *	0.31 **	−0.13	0.24 *	0.19	0.20	0.28 **	0.19	0.26 *	0.03	0.11	0.33 **	1							
15. Total cholesterol (mg/dL)	0.003	0.17	0.27 **	−0.18	0.16	0.11	0.13	0.22 *	0.13	0.23 *	−0.03	0.10	0.24 *	0.74 **	1						
16. Insulin (uIU/mL)	0.21 *	0.37 **	0.19	0.21 *	0.31	0.37 **	0.32 **	0.24 *	0.24 *	0.32 **	0.25 *	−0.18	0.27	0.02	0.04	1					
Spearman Correlations																					
17. Triglycerides (mg/dL)	0.09	0.42 **	0.44 **	−0.01	0.38 **	0.37 **	0.29 **	0.29 **	0.33 **	0.32 **	0.17	−0.17	0.39 **	0.30 **	0.31 **	0.37 **	1				
18. HDL-C (mg/dL)	−0.22 *	−0.43 **	−0.38 **	−0.14	−0.39 **	−0.35 **	−0.35 **	−0.26 *	−0.20	−0.25 *	−0.25 *	0.01	−0.38 **	−0.18	0.10	−0.26 *	−0.37 **	1			
19. Fasting glucose	0.02	0.03	−0.06	0.24 *	0.03	0.03	0.04	0.10	0.00	−0.05	0.19	−0.03	0.01	0.03	−0.02	0.23 *	0.02	−0.05	1		
20. HOMA score	0.25 *	0.38 **	0.24 *	0.19	0.34 **	0.37 **	0.32 **	0.21 *	0.23 *	0.30 **	0.32 **	−0.16	0.29 **	−0.01	0.008	0.91 **	0.30 **	−0.21 *	0.42 **	1	
21. Hepatic steatosis	0.33 **	0.50 **	0.49 **	−0.006	0.47 **	0.46 **	0.49 **	0.29 **	0.30 **	0.47 **	0.29 **	0.09	0.51 **	0.19	0.18	0.17	0.23 *	−0.28 **	−0.07	0.09	1
22. Exercise tolerance	−0.33 **	−0.70 **	−0.67 **	0.06	−0.65	−0.60 **	−0.64 **	−0.39 **	−0.37 **	−0.62 **	−0.30 **	−0.08	−0.71 **	−0.35 **	−0.30 **	−0.25 *	−0.44 **	0.22 *	0.001	−0.18	−0.53 **

Note. * *p* < 0.05; ** *p* < 0.01; N = 88.

**Table 3:** Comparison of selected social and lifestyle-related characteristics between obesity and control groups.

		Obesity Group (*N* = 58)			Control Group (*N* = 30)		
		Percent	Median	IQR	Percent	Median	IQR
Training volume (hours/week)	0–1 h	17.24%	4	6	6.67%	9	7
	2–5 h	44.83%			16.67%		
	6–10 h	22.41%			40%		
	11–15 h	6.89%			26.67%		
	16–20 h	6.90%			6.66%		
	21–25 h	1.72%			3.33%		
Daily screen time (hours/day)	0–1 h	13.79%	3	3	30%	3	2
	2–3 h	41.38%			50%		
	4–5 h	32.76%			16.66%		
	6–8 h	10.35%			3.33%		
	9–10 h	1.72%			-		
Educational level	Kindergarten	3.45%	-	-	3.33%	-	-
	Primary school	34.48%			23.33%		
	Lower secondary school	34.48%			30.00%		
	Upper secondary school	25.86%			43.33%		
Socioeconomic status	Low	13.79%	-	-	16.67%	-	-
	Middle	68.97%			80.00%		
	High	13.79%			3.33%		

## Data Availability

The raw data supporting the information and conclusions of this article will be made available by the authors upon reasonable request, without undue restriction.

## Supplementary Materials

The following supporting information can be downloaded at: https://www.mdpi.com/article/10.3390/diagnostics16081162/s1, Figure S1. Distribution of study cohort by gender (female/male); Table S1. Frequency and percentage distribution of sex and area of residence in the obesity and control groups; Table S2. Descriptive statistics of study cohort; Table S3. Descriptive statistics of biochemical and hematological profile of study cohort.

## Abbreviations

The following abbreviations are used in this manuscript:

ALT	Alanine aminotransferase
AST	Aspartate aminotransferase
BMI	Body mass index
BP	Blood pressure
CI	Confidence interval
COSI	European Childhood Obesity Surveillance Initiative
HbA1c	Glycated hemoglobin A1C
RMR	Resting metabolic rate
SD	Standard deviation
WHO	World Health Organization
