# Supplementary material for: Physical Activity Patterns and Cardiometabolic Risk in Children and Adolescents with Obesity: A Cross-Sectional Study
Source: Diagnostics (Basel). 2026 Apr 14;16(8):1162. doi: 10.3390/diagnostics16081162 (PMC13115053; doi:10.3390/diagnostics16081162)
Supplement: Supplementary file 1 [file diagnostics-16-01162-s001.zip › diagnostics-4217542-supplementary.pdf]

Supplementary Materials

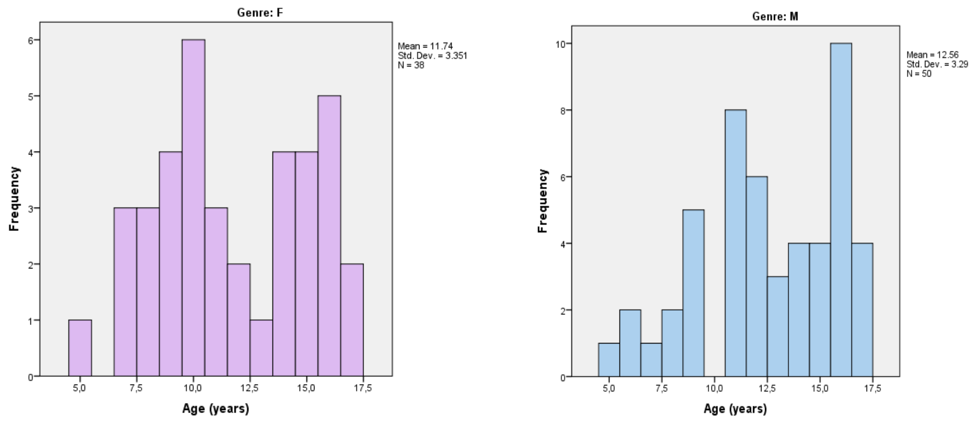

Figure S1. Distribution of study cohort by gender (female/male).

Table S1. Frequency and percentage distribution of sex and area of residence in the obesity and control groups.

Sample		Obesity Group (N = 58)		Control Group (N = 30)	
		Frequency	Percent	Frequency	Percent
Sex	F	21	36,21%	17	56,67%
	M	37	63,79%	13	43,33%
Environment of origin	Urban	28	48,28%	12	40,00%
	Rural	30	51,72%	18	60,00%

Table S2. Descriptive statistics of study cohort.

Descriptive Statistics

	Control Group				Obesity Group			
	Minimum	Maximum	Mean	Std. Deviation	Minimum	Maximum	Mean	Std. Deviation
Age (years)	6	17	12.77	3.319	5	17	11.91	3.315
Weight (kg)	19.0	82.0	45.833	16.0824	34.0	145.0	76.626	26.7218
Height (cm)	113	186	153.80	18.227	117	192	156.07	18.179
BMI (kg/m²)	14,2	26.6	18.733	3.1192	22.4	45.0	30.488	5.3189
Z-Score	-1.82	2.07	-.1160	.83889	1.85	6.28	3.2040	.92636
Abdominal circumference (cm)	49	88	65.50	9.755	72	148	96.12	15.499
Hip circumference (cm)	49	86	71.10	10.420	70	149	99.69	15.029
Waist circumference (cm)	49	84	63.63	8.857	69	140	90.74	14.542
RMR (kcal/day)	945	3265	1375,00	425.074	177	2709	1648.67	435.557
FAT%	3,7	27.0	15.927	6.4081	18.0	43.2	30.864	6.0271
Fat body mass (kg)	.70	16.10	7.6870	3.92848	8.30	62.60	24.6655	11.42153
Lean body mass (kg)	18.30	75.50	38.6787	13.77095	25.20	93.50	51.3116	16.32286
Systolic BP (mmHg)	96	120	106.30	6.834	94	160	118.29	14.056
Diastolic BP (mmHg)	56	90	69.13	8.573	60	110	80.36	9.014
Waist-to-Hip ratio	.64	1.00	.8997	.06631	.76	1.08	.9117	.05629
Waist-to-height ratio	.34	0.52	.4142	.03420	.46	0.77	.5819	.06377

Abbreviations: BMI (Body mass index), BP (blood pressure), RMR (Resting metabolic rate).

Table S3. Descriptive statistics of biochemical and hematological profile of study cohort.

Descriptive Statistics

Sample		N	Minimum	Maximum	Mean	Std. Deviation	Variance	Skewness		Kurtosis	
		Statistic	Statistic	Statistic	Statistic	Statistic	Statistic	Statistic	Std. Error	Statistic	Std. Error
Control Group	Hemoglobin (g/dl)	30	11,10	15,60	13,1567	1,10631	1,224	,433	,427	,042	,833
	Hematocrit (%)	30	32,00	43,70	37,6967	3,02649	9,160	,373	,427	-,462	,833
	Neutrophils (*10³/uL)	30	1,21	12,42	4,7587	2,59689	6,744	1,307	,427	1,633	,833
	Lymphocytes (*10³/uL)	30	,99	47,70	5,5310	10,60396	112,444	3,354	,427	10,803	,833
	Platelets (*10³/uL)	30	193,00	404,00	281,3000	52,86362	2794,562	,533	,427	-,113	,833
	Platelet Distribution Width (fl)	30	8,90	13,10	10,9167	1,00930	1,019	-,034	,427	-,502	,833
	Serum Iron (ug/dl)	30	16,00	144,00	72,5000	25,70489	660,741	,303	,427	1,327	,833
	Erythrocyte Sedimentation Rate (mm/1h)	30	2,00	60,00	7,8000	10,88625	118,510	4,127	,427	19,265	,833
	C-reactive protein (mg/L)	30	,20	149,00	6,6900	26,98112	727,981	5,412	,427	29,495	,833
	Alanine aminotransferase (ALT) (U/L)	30	9,00	58,00	18,9333	10,45824	109,375	2,179	,427	5,835	,833
	Aspartate aminotransferase (AST) (U/L)	30	14,00	50,00	26,4667	9,66948	93,499	1,031	,427	,339	,833
	Urea (mg/dl)	30	17,00	45,00	28,6000	6,38749	40,800	,358	,427	,108	,833
	Creatinine (mg/dl)	30	,45	1,40	,6833	,20083	,040	1,774	,427	4,630	,833
	Uric Acid (mg/dl)	30	2,60	6,40	4,1967	,59509	,354	1,162	,427	7,152	,833
	Glucose (mg/dL)	30	71,00	132,00	89,5000	14,38809	207,017	1,179	,427	1,271	,833
	HbA1c (%)	30	4,00	5,10	4,3433	,26611	,071	1,226	,427	1,625	,833
	Total Cholesterol (mg/dl)	30	112,00	192,00	141,3333	21,15678	447,609	,839	,427	,025	,833
	LDL-C (mg/dl)	30	61,90	121,10	82,0467	17,09857	292,361	1,188	,427	,308	,833
	VLDL-C (mg/dl)	30	9,00	45,00	14,8733	6,88136	47,353	3,196	,427	12,720	,833
	HDL-C (mg/dl)	30	38,00	88,00	54,7333	10,61857	112,754	1,096	,427	1,888	,833
	Triglycerides (mg/dl)	30	50,00	226,00	75,3000	34,40644	1183,803	3,236	,427	12,801	,833
	Total Protein (g/l)	30	59,40	76,60	71,5467	3,83826	14,732	-1,000	,427	2,080	,833
	Insulin (uIU/mL)	30	4,87	91,35	34,7111	22,97905	528,037	1,106	,427	,657	,833
	HOMA score	30	,90	29,80	7,9433	6,14232	37,728	1,858	,427	4,536	,833
	Valid N (listwise)	30									

Obesity Group	Hemoglobin (g/dl)	58	11,60	17,40	13,9121	1,33523	1,783	1,010	,314	,381	,618
	Hematocrit (%)	58	33,50	52,20	41,2414	3,84722	14,801	,661	,314	,194	,618
	Neutrophils (*10^3/uL)	58	1,89	38,90	5,0809	4,72117	22,289	6,662	,314	48,185	,618
	Lymphocytes (*10^3/uL)	58	1,31	11,90	3,0367	1,49818	2,245	3,769	,314	21,186	,618
	Platelets (*10^3/uL)	58	179,00	554,00	330,0690	71,96926	5179,574	,353	,314	,809	,618
	Platelet Distribution Width (fl)	58	8,70	124,00	13,6690	14,84095	220,254	7,459	,314	56,376	,618
	Serum Iron (ug/dl)	58	22,00	143,00	67,9310	27,08911	733,820	,872	,314	,911	,618
	Erythrocyte Sedimentation Rate (mm/1h)	58	2,00	35,00	11,5000	8,27859	68,535	1,134	,314	,567	,618
	C-reactive protein (mg/L)	58	,22	36,69	4,9095	6,29901	39,678	3,003	,314	11,607	,618
	Alanine aminotransferase (ALT) (U/L)	58	11,00	83,00	30,2069	18,08048	326,904	1,270	,314	,732	,618
	Aspartate aminotransferase (AST) (U/L)	58	14,00	84,00	26,4483	11,05199	122,146	2,781	,314	12,296	,618
	Urea (mg/dl)	58	16,00	41,00	27,0345	5,66914	32,139	,392	,314	-,138	,618
	Creatinine (mg/dl)	58	,34	1,10	,5948	,15688	,025	1,060	,314	,993	,618
	Uric Acid (mg/dl)	58	2,90	9,10	5,8500	1,10044	1,211	,237	,314	1,203	,618
	Glucose (mg/dL)	58	67,00	123,00	84,3621	9,58013	91,779	1,162	,314	3,525	,618
	HbA1c (%)	58	4,50	15,00	5,3328	1,31994	1,742	7,137	,314	52,939	,618
	Total Cholesterol (mg/dl)	58	89,00	212,00	157,6379	25,35689	642,972	-,134	,314	,148	,618
	LDL-C (mg/dl)	58	41,00	153,30	101,0741	26,41746	697,882	-,105	,314	-,156	,618
	VLDL-C (mg/dl)	58	6,00	43,00	21,2759	8,81218	77,654	,644	,314	-,290	,618
	HDL-C (mg/dl)	58	24,00	71,00	47,8621	9,34607	87,349	,326	,314	,664	,618
	Triglycerides (mg/dl)	58	29,00	215,00	107,9138	44,10087	1944,887	,622	,314	-,342	,618
	Total Protein (g/l)	58	66,38	82,20	75,0514	3,58865	12,878	-,251	,314	-,383	,618
	Insulin (uIU/mL)	58	9,25	77,33	42,0078	15,80696	249,860	,343	,314	-,194	,618
	HOMA score	58	,30	18,70	8,5914	3,68593	13,586	,288	,314	,534	,618
	Valid N (listwise)	58									
